# Trends in Diagnosis and Treatment of Sacroiliac Joint Pathology Over the Past 10 Years: Review of Scientific Evidence for New Devices for Sacroiliac Joint Fusion

**DOI:** 10.7759/cureus.15415

**Published:** 2021-06-03

**Authors:** Alexander S Himstead, Nolan J Brown, Shane Shahrestani, Katelynn Tran, Jordan L Davies, Michael Oh

**Affiliations:** 1 Department of Neurological Surgery, University of California Irvine, Irvine, USA; 2 Department of Neurosurgery, Keck School of Medicine, University of Southern California, Los Angeles, USA

**Keywords:** sacroiliac joint, arthrodesis, fusion, medical device, minimally-invasive surgery

## Abstract

Sacroiliac (SI) joint pathology is a newly appreciated contributor to lower back pain. Sacroiliac joint fusion (SIJF) has grown rapidly in popularity in association with the advent of minimally-invasive surgical techniques. This has led to an explosion of new medical devices used for SIJF. The objective of this article is to outline clinical trends, summarize the current data, and categorize novel devices for SIJF. Trends in SI joint pathology and fusion were obtained via the Healthcare Cost and Utilization Project’s (HCUP) National Inpatient Sample (NIS) database and Web of Science. To review literature on devices for SIJF, PubMed was searched using the Boolean phrase “sacroiliac joint AND (fusion OR arthrodesis)” since 2010. To establish a list of SIJF devices not represented in the literature, searches were performed on the FDA 510(k), premarket approval, and de novo databases, as well as Google and LinkedIn. Literature review yielded 11 FDA-approved devices for minimally invasive SIJF. Database query yielded an additional 22 devices for a total of 33 devices. Twenty-one devices used the lateral transiliac approach, six posterior allograft approach, three posterolateral approach, and three combined the lateral transiliac and posterolateral approaches. The evidence for the lateral transiliac approach is the most robust. Many novel devices have been developed for minimally invasive SIJF over the past 10 years. Further randomized comparative trials are warranted to evaluate different surgical approaches and novel devices at this time.

## Introduction and background

Lower back pain (LBP) is a significant cause of morbidity worldwide with an annual prevalence ranging from 15-45% [1]. A newly appreciated contributor to lower back pain is the sacroiliac (SI) joint; 15-30% of all LBP is derived from this joint [2, 3]. Treatment for LBP derived from the sacroiliac joint includes physical therapy, corticosteroids, prolotherapy, radiofrequency denervation, and sacroiliac joint fusion [4]. Sacroiliac joint fusion (SIJF) has been around for many years but has grown vastly in popularity over the past 10 years due to the advent of minimally-invasive surgical techniques, new technologies, and improved recognition of the SI joint as a source of back pain [5]. Indeed, in patients with SI joint pathology who fail conservative management, SIJF provides effective pain relief [4, 6], even when compared to optimized conservative management [7-12].

A recent review and meta-analysis on minimally invasive SIJF described two minimally invasive surgical approaches (posterior and lateral transiliac), and six different devices reported in the literature [6]. Of the various devices, the triangular titanium implants (TTIs) (SI-BONE, Inc., Saratoga, CA, USA) have the most substantial evidence [6]. The past 10 years have seen many new devices approved by the United States Food and Drug Administration (FDA), and a majority of these devices have limited clinical research for physicians to use when inferring which device to select. The object of this review is to analyze trends in sacroiliac joint pathology diagnosis, fusion, and literature representation over the past 10 years, and to describe, categorize, and review the evidence for new devices for SIJF to provide clarity on the numerous available options that now exist for this procedure.

## Review

A systemic literature review of PubMed using the Boolean phrase “sacroiliac joint AND (fusion OR arthrodesis)” since 2010 yielded 278 results. Abstracts were reviewed to determine appropriateness. Inclusion criteria consisted of studies on minimally invasive sacroiliac joint fusion using a surgical device on human subjects that looked at improvements in patient-reported outcome measures (PROMs). Exclusion criteria included studies on solely open sacroiliac joint fusion, biomechanical studies, treatment algorithms, and studies in languages other than English. Appropriate manuscripts were downloaded onto Mendeley Reference Manager (Glyph & Cog, LLC, Petaluma, CA, USA). After the initial screen, 154 papers fit the inclusion criteria. Then, each manuscript was reviewed in full and 48 were excluded for a total of 106 manuscripts. These were analyzed to determine which minimally invasive SIJF device was studied. A list of devices represented in the literature was generated. Subsequently, several searches were performed on the FDA 510(k), premarket approval and de novo databases, and the National Institute of Health (NIH) Clinical Trials website, including “SI Joint Fusion”, “Sacroiliac Joint”, “Sacroiliac Joint Fusion”, and “SI Joint”. These searches were repeated on Google and LinkedIn. The sum of these searches produced 33 unique devices. Then, the FDA 510(k) approval documentation and medical device website were referred to for further information about each device, including surgical approach utilized, device composition, predicate devices, and FDA-approved indications.

In addition, the Healthcare Cost and Utilization Project’s (HCUP) National Inpatient Sample (NIS) database was queried using International Classification of Diseases, Tenth Revision (ICD-10) coding for SI joint pathologies and SIJF procedures in 2016. Pathologies included LBP (ICD-10: M545) and SI joint specific pain (ICD-10: M533). Additionally, SIJF procedures were subdivided into open (ICD-10: 0SG704Z, 0SG707Z, 0SG70JZ, 0SH70KZ, 0SG804Z, 0SG807Z, 0SG80JZ, 0SH80KZ) and endoscopic (ICD-10: 0SG734Z, 0SG737Z, 0SG73JZ, 0SG73KZ, 0SG744Z, 0SG747Z, 0SG74JZ, 0SG74KZ, 0SG834Z, 0SG837Z, 0SG83JZ, 0SG83KZ, 0SG844Z, 0SG847Z, 0SG84JZ, 0SG84KZ) approaches. Finally, Web of Science was queried (using the same Boolean phrase as above) to determine recent trends in literature representation of sacroiliac joint fusion.

Results

Trends in Sacroiliac Joint Pathology and Fusion

Using appropriate discharge weights, the 2016 NIS was queried for all SI joint-related pathologies and procedures. A total of 443,505 patients were admitted to a United States hospital for LBP in 2016. LBP specifically in the area of the SI joint was reported in 7,890 patients. As such, 3,210 patients went on to receive an open SIJF procedure and 680 patients received an endoscopic SIJF procedure in 2016.

Until the introduction of ICD-10 coding in the fourth quarter of 2015, SIJF procedures lacked specific International Classification of Diseases, Ninth Revision (ICD-9) codes. In addition, the American Medical Association (AMA) added Current Procedural Terminology (CPT) coding (CPT: 27279) to report procedures involving minimally invasive arthrodesis of the SI joint. The lack of specificity of SIJF coding has prevented the use of nationally representative databases, such as those offered through HCUP, to study SIJF following lumbar and SI joint pathology for many years. With the introduction of ICD-10 procedure coding, contemporary databases may now be used to query cases of SIJF procedures, postoperative complications following SIJF, and indications for SIJF surgery. However, ICD-10 coding still lacks device-specific granularity, preventing comprehensive analysis regarding surgical techniques and fusion materials.

Publications on SI Joint Fusion Per Year

**Figure 1:**
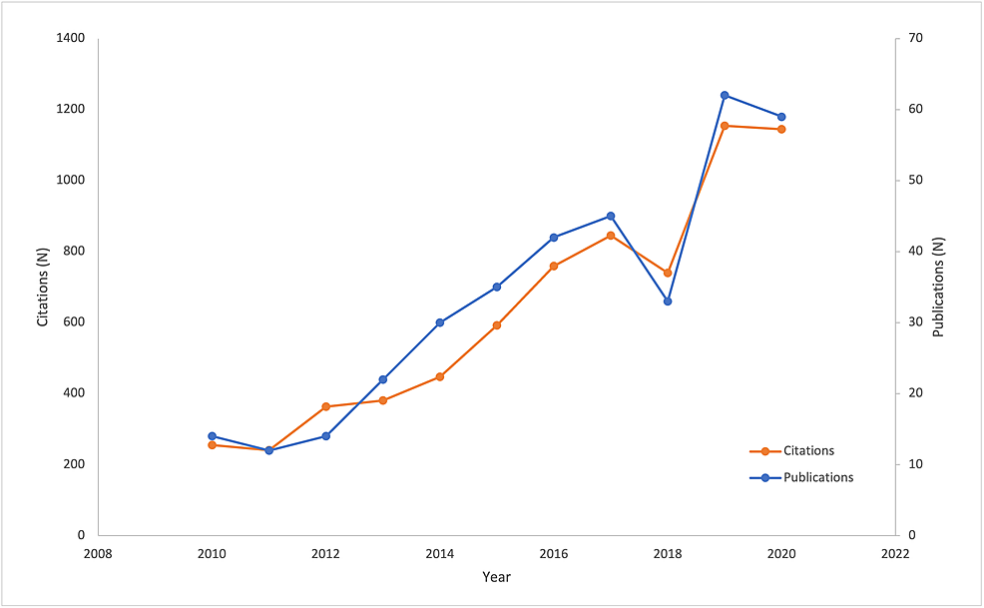
Delineates the upward trend in yearly publications and citations regarding sacroiliac joint fusion over time.

Publications regarding SIJF have trended upward within the past decade (Figure 1). A Web of Science (WoS) search indicated that in 2010, only 14 manuscripts were published on SIJF. By contrast, WoS returned 62 records on SIJF from 2019, which was the peak year for SIJF publications to date. In terms of citations, 2019 saw the greatest number of citations for articles regarding SIJF with 1154 total citations, and 2020 yielded a total citation only slightly lower (1145 citations).

Review of Devices in the Literature

**Figure 2:**
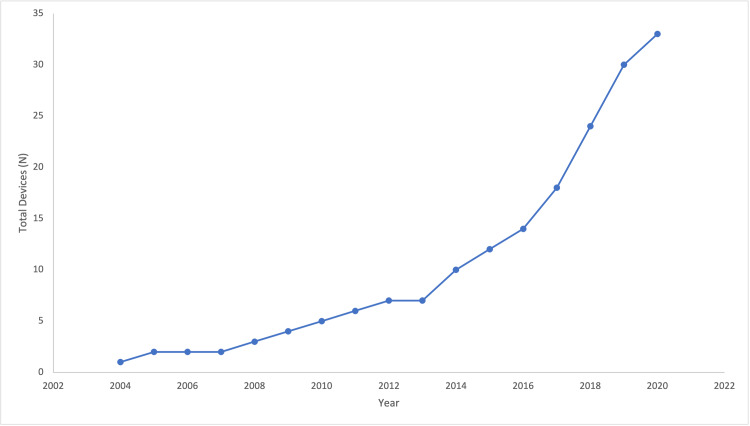
Number of total devices for minimally-invasive sacroiliac joint fusion per year.

**Figure 3:**
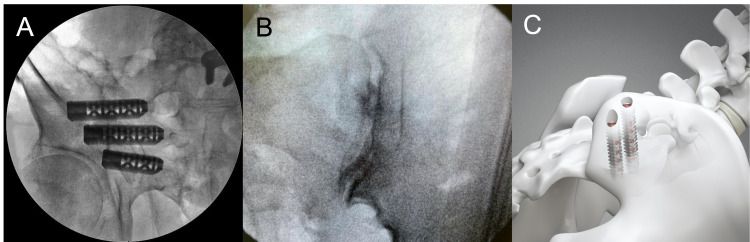
Radiographic or schematic illustrations of the (A) lateral transiliac, (B) posterior inferior, and (C) posterior lateral approaches to sacroiliac joint fusion. Image permissions for re-use were obtained from SI-Bone, Foundation Fusion Solutions, and Medtronic, respectively.

Review of literature yielded 11 unique devices FDA approved for minimally invasive sacroiliac joint fusion (Table 1). Further review of the FDA 510(k), premarket approval, and de novo databases yielded an additional 22 devices with no published literature for a total of 33 devices (Table 2). The increase in total number of minimally invasive SIJF devices by year is displayed in Figure 2. All devices were categorized based on the surgical technique they are designed for. Specifically, 21 devices require a lateral transiliac approach, six devices utilize a posterior allograft approach, three devices employ a posterolateral approach, and three devices combine the posterior lateral with lateral transiliac approaches. The majority of devices using a lateral transiliac approach are a variety of cannulated screw, and the lone exception is the Triangular Titanium Implants (TTIs) manufactured by SI-Bone. The lateral transiliac approach involves inserting up to three implants through the ilium inferior to the sacral ala and anterior to the posterior sacral line (Figure 3A). The posterior allograft approach involves inserting one or two implants, typically filled with bone graft directly into the sacroiliac joint (Figure 3B). The posterolateral approach involves placing typically cannulated screw implants through the ilium and into the sacrum (Figure 3C).

**Table 1:** Devices with published literature, as well as manufacturer, approach, composition, graft compatibility, and evidence. Ltd, Limited; Y, Yes; N, No

Device Name	Manufacturer	Approach	Device Composition	Graft Compatible	Evidence
Titanium Triangular Implants	SI-Bone	Lateral	Triangular Titanium Implants	Y	11 Retrospective Case Series, 4 Prospective Cohort Studies, 2 Safety Analyses
3D Printed Titanium Triangular Implants	SI-Bone	Lateral	Triangular Titanium Implants (3D printed)	Y	1 Case Series, 1 Complaints Analysis
6.5 mm Cannulated Screws	Depuy Synthes	Lateral	Cannulated Screw	N	1 Retrospective Study
Hollow Modular Anchorage Screws	Aesculap Ltd	Lateral	Cannulated screw	Y	3 Prospective Case Series
SI-LOK	Globus Medical	Lateral	Cannulated screw with Hydroxyapatite Coating	Y	1 Prospective Case Series
Firebird Screw System	Orthofix	Lateral	Cannulated Screw	Y	1 Retrospective Case Series
SImmetry	RTI Surgical	Lateral	Cannulated Screw with Bone Decortication	Y	2 Case Series, 1 Level 2 Prospective Trial
Zimmer TriCor	Zimmer Spine	Lateral	Cannulated Screw	Y	1 Case Report
DIANA Cage	Signus Medical	Posterolateral	Hollow Cage	Y	1 Case Report, 1 Retrospective Case Series, 1 Retrospective Comparative Trial
Rialto	Medtronic	Posterolateral	Cannulated Screw	Y	1 Retrospective Case Series, 1 Prospective Observational Study, 1 Case Report

**Table 2:** Devices without published literature, as well as manufacturer, approach, composition and graft compatibility. Ltd, Limited; Y, Yes; N, No

Device Name	Manufacturer	Approach	Device Composition	Graft Compatible
Blue Topaz	Osseus	Lateral	Cannulated Screw	N
Dyna Screw	U&I Corp	Lateral	Cannulated Screw	N
Entasis Dual-Lead Implant	CoreLink	Lateral	Cannulated Screw	Y
EVOL	Cutting Edge Spine	Lateral	Cannulated Screw with Hydroxyapatite Coating	N
Orion	Pantheon Spine	Lateral	Cannulated Screw	Y
Outlet	SIJ Surgical	Lateral	Cannulated Screw	N
PathLoc	L&K Biomed	Lateral	Cannulated Screw	Y
SI-Restore	Biofusion Medical	Lateral	Cannulated Screw	Y
SICure	Alevio Spine	Lateral	Cannulated Screw with Bone Decortication	Y
SILEX	X-Spine Systems	Lateral	Cannulated Screw	Y
SIMPACT	Life Spine	Lateral	Cannulated Screw	N
UNITY	Huvexel Co., Ltd	Lateral	Cannulated Screw	Y
M.U.S.T. SI Screw System	Medacta International	Lateral	Cannulated Screw with Hydroxyapatite Coating	N
Catamaran	Tenon Medical	Posterior allograft	Double-Barreled Hollow Metal Allograft	Y
CornerLoc	Fusion Foundation Solutions	Posterior allograft	Bone Allograft	N
LinQ	PainTEQ	Posterior allograft	Bone Allograft	N
Posterior Si Fusion System	Omnia Medical	Posterior allograft	Bone Allograft	N
SIFix System	Nu Tech	Posterior allograft	Bone Allograft	N
TransFasten	Captiva Spine	Posterior allograft	Bone Allograft	N
Sacrix Sacrofuse System	KIC Ventures	Posterolateral	Cannulated Screw	N
Genesys Spine	Genesys Spine	Posterolateral- Lateral Combined	Cannulated Screw	Y
Prolix/Siconus	Camber Spine Technologies	Posterolateral- Lateral Combined	Bone Allograft (Prolix) with Cannulated Screws (Siconus)	N
SIJFuse	SpineFrontier Inc.	Posterolateral- Lateral Combined	Cannulated Screw	Y

United States Food and Drug Administration Approval

Out of 33 devices, 24 were approved by the FDA or Conformitè Europëene (CE) on the basis of substantial equivalence to one or more predicate devices. Of the nine devices without FDA 510(k) substantial equivalence determination, the majority (five) employed the posterior allograft approach, while two were posterolateral and two used the combined approach. There were 10 total primary predicates. The most commonly cited was the 6.5mm Cannulated Screw (Depuy Synthes, West Chester, PA, USA; cited six times as primary predicate, eight times total). All device indications included “Sacroiliac joint fusion for conditions including sacroiliac joint disruptions and degenerative sacroiliitis”, except for the locking cannulated screw (U&I Corp., Uijeongbu, South Korea), which had several additional joint fusion-related indications. The sacroiliac joint fusion solution by Foundation Fusion Solutions, LLC (Tulsa, OK, USA) is the sole device of the nine that is currently undergoing a clinical trial, identified on the National Institute of Health Clinical Trials website.

Summary of Evidence

The lateral approach to sacroiliac joint fusion has the largest number of devices and the most robust body of evidence. A recent meta-analysis summarizes this literature well [6], but we will provide a brief overview. Much of the evidence for lateral SIJF is derived from research on the triangular titanium implants, which are composed of three triangular titanium hollow porous rods that are inserted across the SI joint (Figure 3A). This device may be used with bone graft and a 3D-printed version of this device has recently been developed. Combining regular and 3D-printed TTIs, the evidence for the triangular titanium implants consists of over 19 studies. These include 11 retrospective case series (level 4 evidence) [13-23], two level 2 prospective cohort studies [24, 25], two level 1 prospective studies [8, 10], as well as one case series on a 3D-printed version of the implant [26].

In summary, a total of 629 (605 traditional TTIs, 24 3D-printed) patients were treated with the TTIs across these studies. The follow-up and validated outcome measures used in the retrospective case series were variable, but the prospective studies uniformly report improvements in preoperative to postoperative Oswell Disability Index (ODI), decreased need for opioids, low surgical revision rate, low adverse event rate, and improved functionality. Additionally, the device manufacturer published two safety analyses from internal databases on a total of 11,388 procedures [27, 28]. These articles report an overall low rate of complaints (3.8%) and high implant survivorship (96.46%) at four years with the TTI system. A safety analysis on the 3D-printed triangular titanium implants found no difference in complaint or complication rate between 3D-printed vs. traditional TTIs [27].

SImmetry Sacroiliac Joint Fusion System (RTI Surgical, formerly Zyga Technology, Alachua, FL, USA) is the lateral device with the next highest level of evidence consisting of two case series (level 4 evidence) and one level 2 prospective cohort study on a total of 87 individuals [29-31]. This system employs a similar insertion trajectory as TTIs, but a tool is used to decorticate the surface of the sacroiliac joint prior to insertion of the cannulated screw with bone graft. Studies on this technology report significant improvements in baseline to postoperative pain and ODI scores. Notably, Kube and Muir found that only eight of 18 patients achieved clinically significant improvement in ODI [31]. Six total adverse events related to the device were reported, including one requiring a revision procedure for nerve impingement.

The 6.5mm Cannulated Screw by Dupuy-Synthes is used in lateral SIJF, and is the most commonly listed primary predicate for new devices. It is listed as predicate device for 10 devices, including the TTIs (SI-Bone), the hydroxyapatite coated screw (SI-LOK, Globus Medical, Audubon, PA, USA), the threaded titanium implant (SImmetry), the outlet sacroiliac joint fusion system (SIJ Surgical, Raleigh, NC, USA), the PathLoc SI Joint Fusion System (L&K BioMed Co., Ltd., South Korea), the UNITY sacroiliac joint fixation system (Huvexel Co., Ltd, South Korea), hollow metal double-barreled posterior allograft device (Catamaran; Tenon Medical, Inc., San Ramon, CA, USA), Dyna locking cannulated screw (U&I Corp), Entiasis dual-lead sacroiliac implant (CoreLink, LLC, St. Louis, MO, USA) and SILEX (X-Spine Systems, Inc., Miamisburg, OH, USA). The evidence supporting the 6.5mm Cannulated Screw as primary predicate is one retrospective comparative study, which compared 38 screw-based SI joint fixation with 274 using TTIs and found an increased surgical revision rate in the screw-based fixation group compared to the TTI fusion group (30.8% vs. 5.7%) [32]. The authors did not report pain relief or disability improvement between groups. Of note, this study was funded by SI-Bone.

The posterior allograft approach (Figure 3B) was employed in one study by Wise and Dall in 2008 [33]. This prospective study on 13 consecutive patients found significant improvements in final low back pain score on a visual analog scale (VAS), with an average improvement of 4.9 (p < 0.001), with no infections or neurovascular complications and an overall fusion rate of 89% six months postoperatively [33]. They described their device as a “threaded titanium cage” by Medtronic used off-label, and it appears different from Medtronic’s current SIJF device, with the trade name “Rialto”. The only posterior allograft device that is supported by FDA 510k substantial equivalence determination is a hollow metal double-barreled posterior allograft implant by Tenon Medical (San Ramon, CA, USA), and lists Medtronic’s SIJF device as its primary predicate device. The Tenon Medical website does report recently finishing a post-approval clinical trial that found greater than 85% SI joint pain reduction at six weeks after surgery, and radiographic evidence of fusion at six months, but does not provide further information regarding study design or results [34]. None of the other five posterior allograft devices required an FDA 510(k) substantial equivalence determination, because they involve placing only bone allograft into the joint space, which does not require FDA approval.

Posterolateral devices are placed using a minimally invasive approach to broach the sacroiliac joint from the posterior superior iliac spine anteroinferiorly (Figure 3C). The cylindrical threaded implant by Medtronic is the object of one case report, one retrospective case series (n = 24), and one retrospective comparative study vs. TTIs (n = 74 vs. 82) [35-37]. The case series reported mean total satisfaction score of 89.0% ± 27.6%, with a statistically significant reduction in postoperative low back pain score (6.6 ± 2.4 to 3.7 ± 3.3) and leg pain score (4.8 ± 3.8 to 1.5 ± 2.9), compared to preoperative pain. The retrospective comparative study found shorter operation time (41.2 min vs. 60.0 min) and insignificantly reduced complication rate (2.4% vs. 6.1%) for TTIs, and similar improvement in patient-reported outcomes (PROs) including postoperative visual analog scale (VAS), Oswestry Disability Index (ODI) and Short Form-12 at one year follow-up.

The hollow cage by Signus Medical (trade name DIANA Cage, Washington D.C., USA) is a hollow cage or dowel-based system that utilizes the posterolateral approach. Evidence includes one retrospective case series (n = 19), and one large, multicenter prospective observational study involving 20 hospitals in Germany (n = 171) [38, 39]. The case series found decreased SI joint pain scores (8.5 to 6) from before surgery to 13-month follow-up. Radiographic evidence of fusion was seen in 79% of joints [39]. The prospective observational study reported encouraging improvements in multiple patient-reported outcome measures (PROMs), including visual analogue score (VAS) (74 to 37) and Oswestry Disability Index (ODI) (51 to 33) [38]. The sacroiliac joint fusion rate at 13-month CT scan was 31%.

None of the combined posterolateral and lateral devices have published literature. These devices work primarily by inserting a bone allograft posteriorly into the sacroiliac joint (posterolateral approach) and fastening it in place by one or more screws placed through the bone allograft (lateral approach).

Discussion

Sacroiliac joint pathology has been increasingly recognized as a contributor to lower back pain, and sacroiliac joint fusion to treat this condition has grown in popularity over the past 10 years [5]. Yearly publications regarding SIJF have increased, number of citations have increased, and a large number of patients are diagnosed with SI joint pathology and subsequently underwent fusion in 2016. The increased recognition and popularity of SI joint pathology around 10 years ago coincides with the FDA approval of the triangular titanium implant device for sacroiliac joint fusion in 2008, and subsequent studies validating its efficacy and cost-effectiveness in 2013 and 2014 [6]. It is possible that the establishment of this technology as safe, efficacious, and profitable stimulated rapid development of novel devices for SIJF seen over the past 10 years (four devices in 2010 to 33 in 2020) (Figure 2).

As described in this review, the triangular titanium implants have the most robust literature support, and have been shown to be a safe, effective means of sacroiliac joint fusion. The strongest support for the TTIs is the SIFI (NCT01640353) and INSITE (NCT01681004) trials, which were large, multicenter, randomized prospective cohort trials. Many new devices utilize a similar lateral transiliac approach to joint fusion. However, they are mostly screw-based and most cited the 6.5mm cannulated screw (Dupuy-Synthes) as a predicate. This is potentially problematic, because the only study on the use of the 6.5mm cannulated screw in SIJF was a comparative study with a small sample size (n = 38) and was funded by the manufacturers of the TTIs. Further contributing to bias, authors only reported surgical revision rate [32]. Based on the limited evidence, it appears that many of the new lateral transiliac devices remain unproven. More work is required to validate the safety and efficacy of these devices in SIJF.

The posterolateral approach has the theoretical benefit of minimizing tissue disruption and requiring fewer implants, which may reduce operative time (Figure 3C). One retrospective cohort study compared TTIs with cylindrical threaded implants (Rialto, Medtronic, Memphis, TN, USA) and found longer operative times with the cylindrical threaded implants, which use the posterior approach [36]. However, the authors attributed this to the use of stereotactic navigation in the posterior implant group, which increased total time due to longer pre-operative and post-operative imaging. Furthermore, the surgeons were less experienced with the new posterolateral procedure, which has been shown to increase operating times [40-44]. In addition, the study showed a statistically significant decrease in estimated blood loss in the posterior group, and a non-significant increase in revision rate in the posterior group [36], which supports that cylindrical threaded implants are a reasonable choice for SIJF. The study found no significant difference in PROMs (pain, quality of life, disability). Devices that combine posterolateral and lateral fusion may provide more secure and longer-lasting fusion, as the lateral devices lock the posterolateral implant in place, but this is speculative as there have not been trials comparing combined devices with either lateral or posterolateral devices alone.

Posterior allograft devices are the most unique, consisting of one or two spacers inserted into the SI joint posteriorly (Figure 3B). Interestingly, as these devices qualify as bone allograft, they did not require an FDA 510k substantial equivalence determination. Implantation of these devices is likely the simplest as much less tissue disruption is required. Furthermore, these devices can be placed by pain physicians and do not require an experienced neurosurgeon or orthopedic surgeon. Based on their action as spacers that sit in the joint rather than devices crossing the joint to lock it in place, these theoretically may have shorter longevity and higher revision rates compared to devices utilizing the posterolateral or lateral transiliac approaches, although no studies have been done to verify this. If these devices provide adequate pain relief for any period of time and do not cause significant side effects, they may be utilized in a manner analogous to glucocorticoid injections for joint pain, as a preliminary treatment strategy to surgical fusion.

There are a number of unique qualities between devices within the same category that may contribute to clinical outcomes. The decortication and fixation SIJF system (SImmetry, RTI Surgical) is the only device that utilizes a decortication and fixation process to achieve joint fusion. This method is currently under study in a five-year industry-sponsored trial that completed data collection in 2019 but is not yet published (Evolusion Study, NCT02074761).

At this point, there are numerous devices for sacroiliac joint fusion, and despite many potential benefits and drawbacks of each system, further clinical research is required to develop more definitive guidelines for when different device types are indicated. While more evidence is required to evaluate the relative superiority of one device over the others and provide definitive recommendations, the triangular titanium implants and cylindrical threaded implants boast the most robust evidence, and either may be used depending on the surgeon’s preference. Regardless of the system used, it is the responsibility of the medical device company to produce unbiased clinical research validating that their novel device works as indicated in human subjects. At the same time, it is the responsibility of the physician to evaluate the current literature and utilize the device that is most validated to produce high-quality outcomes.

## Conclusions

Minimally invasive sacroiliac joint fusion is an increasingly popular procedure for treatment of chronic refractory low back pain isolated to the SI joint. Many novel and different devices have been developed for this indication over the past 10 years. While the triangular titanium implants and cylindrical threaded implants have the most robust literature backing their efficacy, the majority of novel implants are varieties of a cannulated screw. Further randomized comparative trials that investigate different aspects of each novel device is warranted to evaluate novel devices and elucidate unique features that may be of clinical benefit.
